# The Effect of Diagonal Exercise Training for Neurorehabilitation on Functional Activity in Stroke Patients: A Pilot Study

**DOI:** 10.3390/brainsci13050799

**Published:** 2023-05-14

**Authors:** Jung-Ho Lee, Eun-Ja Kim

**Affiliations:** Department of Physical Therapy, Kyungdong University, 815, Gyeonhwon-ro, Munmak-eup, Wonju-si 26495, Gang-won-do, Republic of Korea; ljhcivapt@naver.com

**Keywords:** neurorehabilitation, stroke, diagonal exercise training, functional activity

## Abstract

Functional movements of the human body occur multifacetedly. This pilot study investigated the effects of neurorehabilitation training, including diagonal movements, balance, gait, fall efficacy, and activities of daily living in stroke patients. Twenty-eight patients diagnosed with stroke by a specialist were divided into experimental groups applying diagonal exercise training and control groups applying sagittal exercise training. The five times sit-to-stand test (FTSST), timed up and go (TUG) test, and Berg balance scale (BBS) were used to evaluate balance ability, the falls efficacy scale (FES) was used to evaluate fall efficacy, and the modified Barthel index (MBI) was used to evaluate activities of daily living. All evaluations were conducted once prior to intervention implementation and again six weeks after the final intervention. In the study results, the experimental group to which the diagonal exercise training was applied had statistically significant changes in FTSST, BBS, and FES compared to the control group. In conclusion, the rehabilitation program, including diagonal exercise training, increased the patient’s balance and reduced the fear of falling.

## 1. Introduction

A stroke is one of the cerebrovascular diseases that generally refers to a situation where damage occurs in brain tissue due to the occlusion or rupture of cerebral blood vessels. This situation typically arises from the internal coagulation of blood or damage to the vessel wall. The most common cause of stroke is a lack of blood supply due to the obstruction or rupture of blood vessels, such as thrombosis or cerebral hemorrhage. This lack of blood supply interferes with the oxygen and nutrient supply to brain cells, leading to brain tissue damage [1]. The mechanism of stroke is determined by various factors that interact with each other, typically including high blood pressure, which is one of the major risk factors for stroke that causes damage to the vessel wall and promotes blood coagulation, increasing the risk of stroke. Other factors, such as an abnormal increase in platelet count or platelet dysfunction, can also increase the risk of blood coagulation and promote stroke. In addition, damage to the vessel wall, arteriosclerosis, diabetes, and other conditions are known as factors contributing to the development of stroke [2].

Stroke is one of the most common neurological disorders that require long-term rehabilitation therapy after occurrence. Stroke patients usually lose their independence in daily life and are affected by various functions such as movement, cognition, and language [3]. In particular, balance impairment that occurs after a stroke causes significant inconvenience in daily life and is one of the essential tasks in rehabilitation therapy. Balance impairment can lead to problems in walking, performing daily activities, sports, and leisure activities, ultimately lowering the patient’s quality of life [4].

The disabilities caused by stroke are very diverse, including motor, cognitive, language, sensory, and perceptual function impairments. These disabilities can greatly reduce the quality of life of patients and also burden their families and society. Motor function impairment is one of the most common disabilities after a stroke, usually resulting from cerebral infarction caused by stroke, and patients experience symptoms such as hemiplegia or hemiparesis. These symptoms lead to a decline in motor function, causing problems in walking, performing daily activities, and balance control [5]. Cognitive function impairment can also occur after a stroke, and this can manifest as a decline in concentration and memory for cognitive function. In addition, patients may have difficulty understanding conversations or comprehending other people’s speech, which can cause significant problems in daily life [6].

Stroke can cause neurological symptoms that affect both the peripheral and central nervous systems, and falls are one of the most common issues among stroke patients. Falls occur when stroke patients are unable to maintain a stable posture and lose their balance, which can be a major problem in their daily lives. Stroke patients often experience decreased balance and are more prone to falls in their daily activities [7]. Furthermore, falls can lead to injuries that prolong the rehabilitation period or even worsen the condition to the point where they are unable to carry out their daily activities. Additionally, falls can lower a stroke patient’s self-confidence and lead to various negative effects, such as loss of social relationships. Therefore, proactive measures to prevent falls are necessary for stroke patients [8].

The walking ability of stroke patients is related to changes in their walking patterns. Walking is a complex movement involving the coordinated motion of the legs, pelvis, torso, muscles, and more [9]. However, brain damage due to stroke can reduce the coordination of walking patterns and cause problems with movement control. Stroke patients often experience problems with walking speed, walking symmetry, weight shifting, and foot support time, and these problems increase the risk of falling along with decreased walking ability. Stroke patients typically have a slow and unstable walking pattern, which can limit their daily activities and cause social impairments. Therefore, active measures are necessary to prevent walking disorders and improve walking ability, such as appropriate rehabilitation programs and walking training. Through these interventions, stroke patients can expect to recover their walking ability, improve their walking patterns, and reduce the risk of falling [10].

Activities of daily living (ADL) refer to the everyday activities that individuals perform in their daily lives, including eating, bathing, using the toilet, and getting dressed [11]. Stroke patients often experience difficulties in performing these ADLs, which can decrease their quality of life. Stroke causes damage to specific areas of the brain, which impairs the ability to perform daily activities. In particular, stroke has a significant impact on everyday movements such as balance, walking, and the movement of the wrists and hands. This is directly linked to a decrease in ADL function among stroke patients [12].

Stroke treatment can be broadly divided into medication therapy and rehabilitation therapy. Medication therapy involves the use of drugs such as thrombolytics, anticoagulants, and antihypertensives to improve cerebral blood flow and promote circulation [13,14]. Rehabilitation therapy focuses on improving physical and cognitive function following stroke and is crucial for enhancing functional recovery and performing activities of daily living. Rehabilitation therapies include gait training, strength exercises, and cognitive training, among others, and are designed to improve the motor and cognitive impairments associated with stroke and enhance the patient’s ability to perform daily activities [15,16].

Proprioceptive neuromuscular facilitation (PNF) is a type of neuro-muscular rehabilitation therapy that improves muscle movement patterns to enhance strength and balance. PNF diagonal movements utilize diagonal movements between the upper and lower extremities to improve muscle control, increase strength, and enhance balance [17]. In stroke rehabilitation, PNF diagonal movements are effective for gait and balance rehabilitation, improving upper and lower extremity coordination, enhancing gait stability, and improving balance. Additionally, PNF diagonal movements are useful for training activities of daily living, improving the patient’s ability to perform ADLs, and promoting independence in daily life [18]. PNF therapy distinguishes itself from other therapeutic approaches through the use of specific patterns and techniques that target the body’s proprioceptive system. The proprioceptive system, composed of sensory receptors within muscles, tendons, and joints, is responsible for providing the brain with feedback about the body’s position, movement, and force. By targeting this system, PNF therapy can effectively address various neuromuscular and musculoskeletal issues [19].

The effectiveness of PNF therapy is rooted in a variety of theories and principles, from neurophysiology to biomechanics and motor learning. These key concepts form the foundation of PNF therapy and contribute to its success in addressing various neuromuscular and musculoskeletal issues. One critical concept in PNF therapy is autogenic inhibition, which refers to the reflexive relaxation response that occurs in a muscle after it has undergone a sustained contraction. PNF techniques, such as contract–relax and hold–relax, take advantage of this principle to facilitate an increase in muscle length and enhance flexibility [20]. Reciprocal inhibition is another important principle utilized in PNF therapy. This concept involves the relaxation of one muscle group while the opposing muscle group contracts, leading to an augmented stretching experience and improved range of motion. The interaction between these muscle groups allows for more effective stretching and greater overall flexibility [21]. Additionally, PNF therapy incorporates principles of motor learning, including repetition, feedback, and task specificity. By integrating these elements, therapy promotes the development of new motor patterns and the fine-tuning of existing ones. This focus on motor learning ultimately results in better functional movement and improved physical performance [20].

Diagonal movement in the human body refers to movements where the upper and lower extremities move diagonally. These movements have different patterns compared to sagittal plane movements and play an important role in various functions of the human body. Research has shown that diagonal movements use more muscles and are more effective in improving muscle strength and balance compared to sagittal plane movements [22]. Diagonal movements connect muscles in the upper and lower extremities, thus improving muscle strength and also enhance balance by stimulating the balance sensory organs in the brain, such as the vestibular system. Diagonal movements are frequently used in daily activities such as walking, climbing stairs, and dressing, making them important for improving overall functional ability in daily life [23,24]. However, many current rehabilitation methods in clinical practice focus on single joint movements rather than utilizing movements that involve the entire body. 

The primary objective of this study is to evaluate the effectiveness of PNF-based diagonal exercises in stroke rehabilitation therapy on improving balance and walking abilities in stroke patients compared to the sagittal exercise rehabilitation methods. The second aim of this study is to explore the influence of PNF-based diagonal exercises on addressing the psychological aspect of fear of falling and to evaluate its impact on the performance of activities of daily living in stroke patients.

## 2. Materials and Methods

### 2.1. Subjects

In this study, 28 patients who were diagnosed with stroke due to cerebral infarction by a rehabilitation medicine specialist based on evaluation using diagnostic imaging equipment among patients visiting the rehabilitation department were selected as study subjects. The study subjects were selected based on the following inclusion criteria: patients within 12 months of stroke onset, not taking medication to reduce spasticity, experiencing hemiplegia on one side, able to walk independently without an assistance device, having no history of fractures or falls, possessing a mini-mental state examination (MMSE) score of 24 or higher, and understanding the study content while being capable of performing exercises and assessments as per the therapist’s instructions. The exclusion criteria for study subjects included secondary stroke patients, patients unable to balance due to cerebellar lesions, those receiving treatment for orthopedic diseases, patients taking antidepressants, and individuals with severe synergistic patterns in the lower extremities that prevent knee flexion. All subjects were given a detailed and general explanation of the experiment, including all purposes, procedures, methods, limitations, and patients’ rights, before the experiment and were allowed to participate in the research after signing the consent form in their handwriting. In addition, all research processes were conducted in accordance with the Declaration of Helsinki and conducted under the supervision of the Research Ethics Committee.

### 2.2. Designs

**Figure 1:**
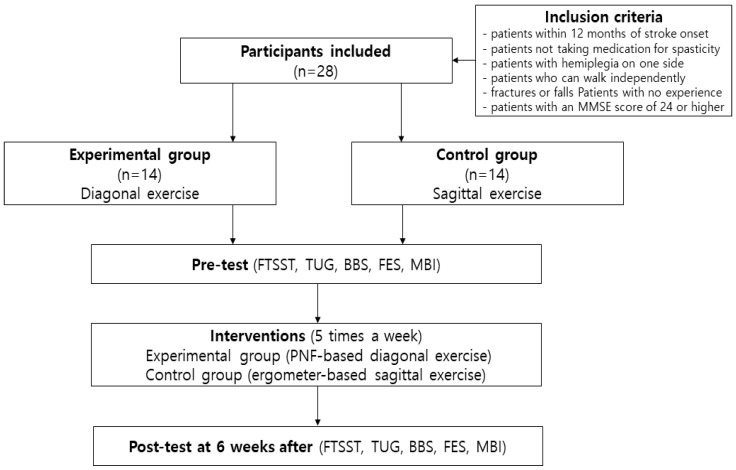
Flowchart of study.

The study subjects were assigned to either an experimental group or a control group. The patients who selected the “O” card were assigned to the experimental group, while those who selected the “★” card were assigned to the control group. The experimental group (EP) consisted of 14 patients who performed upper and lower extremity diagonal exercises using PNF for 30 min after receiving general rehabilitation treatment. The control group (CO) also consisted of 14 patients who performed upper and lower extremity cycles for 30 min for sagittal plane exercise after receiving general rehabilitation treatment. All therapeutic interventions were conducted 5 times a week for a total of 30 sessions over a period of 6 weeks. All evaluations were conducted once prior to intervention implementation and again six weeks after the final intervention (Figure 1).

### 2.3. Experimental Procedures

#### 2.3.1. Diagonal Exercise Using Proprioceptive Neuromuscular Facilitation Therapy

In this study, the experimental group performed upper and lower extremity diagonal exercises using PNF. PNF patterns involve diagonal movements that simultaneously activate muscles in different directions and have been shown to be effective in improving muscle strength, muscular endurance, balance, and fine motor control. The upper limb patterns used in this study were the D1 and D2 patterns. The D1 flexion pattern starts with shoulder joint extension–abduction–internal rotation and ends with shoulder joint flexion–adduction–lateral rotation. The D1 extension pattern starts with shoulder joint flexion–adduction–lateral rotation and ends with shoulder joint extension–abduction–internal rotation. The D2 flexion pattern starts with shoulder joint extension–adduction–internal rotation and ends with shoulder joint flexion–abduction–lateral rotation. The D2 extension pattern starts with shoulder joint flexion–abduction–lateral rotation and ends with shoulder joint extension–adduction–internal rotation. For each PNF pattern applied to the upper extremity of the experimental group, 10 repetitions were defined as 1 session, and a total of 3 sessions were applied. A break of 1 min was applied between each PNF pattern.

To apply the PNF lower extremity pattern in this study, the subject placed their lower extremity on the paretic side in the 7 o’clock direction while in the supine position. The starting position of the pattern involved hip extension–abduction–internal rotation, knee extension, ankle plantar flexion, and the arms resting comfortably beside the trunk. To begin the D1 flexion pattern, the therapist instructed the subject to perform ankle dorsiflexion, knee flexion, and hip joint flexion–adduction–lateral rotation, which are opposite motions to the starting posture. After completing the D1 flexion pattern, the therapist performed the D1 extension pattern by changing the position of the hand. To apply the lower extremity D2 flexion pattern, the starting posture was hip extension–adduction–internal rotation and the final posture was hip flexion–abduction–lateral rotation. The therapist changed the position of their hand to apply the D2 extension pattern. During the application of the PNF pattern, the therapist used the minimum resistance that the patient could overcome. All treatment was performed by two physical therapists with more than 10 years of clinical experience.

#### 2.3.2. Sagittal Exercise Using Upper and Lower Limb Ergometers

In this study, upper and lower limb ergometers from medBike^®^ (Biodex Medical Systems, Shirley, NY, USA) were used to provide sagittal motion. Upper and lower extremity ergometer exercise is a type of exercise that strengthens upper and lower body muscles and improves cardiovascular function using the hands, arms, hip joints, and knees. This exercise was applied to patients in the control group of this study. During the exercise using the upper and lower limb ergometer, the exercise intensity was gradually increased according to each individual patient’s ability, starting at 20% of their remaining heart rate. Each patient had a 5 min warm-up and recovery exercise time. For patients who were unable to grip the handle during the exercise using the upper extremity ergometer due to muscle contracture or weakness, a Velcro-attached wrist brace was used to fix the wrist and fingers while holding the handle. For patients with severe paralysis, a support was applied at the elbow joint to protect the shoulder joint in the part where the shoulder joint flexion was rapid. For patients with difficulty in flexion of the knee and hip joints with the synergistic pattern of the lower extremity, the lower extremity on the affected side was excluded, or the rotation speed was lowered to accommodate their abilities.

### 2.4. Assessment Methods 

#### 2.4.1. Balance 

The five times sit-to-stand test (FTSST) is a physical performance measure that is commonly used in clinical settings to assess lower body strength, balance, and functional mobility. It involves measuring the time it takes a person to stand up from a seated position and then sit back down again. During the test, the individual sits in a chair with their arms crossed over their chest and their feet flat on the floor. They are then asked to stand up from the chair as quickly as possible and fully extend their legs before sitting back down again. The test is repeated three times, and the average time is calculated. The sit-to-stand test can be used to evaluate a person’s physical function, particularly in older adults or those with mobility impairments. A longer time to complete the test may indicate lower body weakness or poor balance and coordination, which could increase the risk of falls or limit daily activities. It can also be used to monitor changes in physical function over time and to track the effectiveness of interventions aimed at improving mobility and strength [25].

In this study, the TUG (timed up and go) test was conducted to assess the patient’s balance and walking ability. The TUG test is a quick and simple method for evaluating the balance and functional mobility of patients with nervous system damage or the elderly. To conduct the TUG test, the patient was seated comfortably in a stable chair with back support, with their feet placed flat on the floor and their knees and toes not touching each other. The examiner then gave the command for the patient to stand up from the chair as quickly as possible, walk, and then sit back in the chair. The result value of the test reflects the patient’s functional movement, with a smaller value indicating better functional movement and a larger value indicating slower walking speed, poorer balance, and lower functional independence. In this study, three evaluations were conducted, and the average value was used as the measured value [26].

The Berg balance scale (BBS) is a standardized tool used to assess gait and balance function. It consists of 14 items and assesses the functional activities that participants are able to perform in their daily life. Each item is evaluated on a 5-point scale ranging from 0 to 4 points, with a total score of 56 points. The BBS is widely used to evaluate the gait and balance functions of people with functional decline, such as patients with central nervous system damage and the elderly. A BBS score of 20 or less indicates a high-risk group, a score of 21–40 indicates a medium-risk group, and a score of 41–56 indicates a low-risk group. The high-risk group has a high risk of falling and needs to improve gait and balance, while the medium-risk group may have some limitations in performing activities of daily living, so intervention strategies may be required. The low-risk group means that there is no problem with activities of daily living. Currently, the BBS is used in clinical practice to evaluate walking ability, balance maintenance ability, and activities of daily living not only in healthy people but also in studies targeting people with functional declines such as paralysis, spinal cord injury, muscular dystrophy, and the elderly [27].

#### 2.4.2. Confidence in Falling 

The falls efficacy scale (FES) used in this study is a tool that measures confidence in falling and consists of 16 items. Each item is scored on a scale of 0 to 10, where a score of 0 means “not at all confident” and a score of 10 means “complete confidence”. The total score of the FES is the sum of the scores for each item, with a perfect score of 160. A total FES score of 0–19 indicates a very low level of self-confidence, with the person feeling restricted in their daily life movements and experiencing constant anxiety and fear. A score of 20–27 indicates a low level of confidence, but the person is still able to maintain confidence while performing daily activities. A score of 28–31 indicates a moderate level of confidence, with the person maintaining confidence in their daily life and not feeling restricted in walking. Therefore, a lower FES score indicates a higher fear of falling. The KFES is a useful tool for assessing confidence in falling and can provide insight into a person’s daily life movements and level of anxiety related to the risk of falling [28].

#### 2.4.3. Activities of Daily Living

In this study, the modified Barthel index (MBI) was used to evaluate activities of daily living (ADL). The Barthel index is a tool used to evaluate the daily living activities of post-stroke patients by classifying activities of daily living into 10 items. These items include personal hygiene, dressing, using the toilet, controlling the bowel and bladder, moving from a bed to a chair and vice versa, walking, climbing stairs, picking up objects, eating, and drinking. Each item is evaluated, and a score is given for each item. The Barthel index is derived by summing up these scores. A total score of 60 or more on the Barthel index indicates the ability to perform independent daily living activities, while a score of 40 or less indicates severe restrictions on activities of daily living. The Barthel index is a useful tool in assessing a patient’s ability to perform activities of daily living and can provide insight into the degree of independence and functional impairment after stroke [29].

### 2.5. Statistical Analysis Method

In this study, the general characteristics and dependent variables were analyzed using the SPSS 18.0 (SPSS Inc. Chicago, IL, USA) version program. The normality distribution of each variable was confirmed using the Shapiro–Wilk test and statistical analysis was performed using the parametric statistics method. Descriptive statistics were used to calculate the mean and standard deviation, while an independent *t*-test was used to test the homogeneity of the two groups. To evaluate changes within groups over time, a paired *t*-test was conducted. To examine differences between the experimental and control groups during the evaluation period, an independent *t*-test was conducted. The significance level was set at α < 0.01.

## 3. Results

### 3.1. General Characteristics of Subjects and Homogeneity Test of Pre-Test

The general characteristics of the subjects and the descriptive statistics for the evaluation items are shown in Table 1, and in the independent sample *t*-test used for the homogeneity test, the *p*-value for all variables was greater than 0.05, so there was no significant difference between the two groups.

### 3.2. Clinical Outcomes of the Experimental Group and Control Group 

The results of the experimental group’s functional outcomes at baseline (T1) and after 6 weeks (T2) are presented in this study. The data, including the mean ± standard deviation (SD) and the corresponding t and *p* values from the paired *t*-test, indicate significant improvements in all functional outcomes for the experimental group after the 6-week intervention (Table 2). 

The experimental group demonstrated a significant decrease in the mean score for the five times sit-to-stand test from 17.78 s (±2.54) at T1 to 13.85 s (±2.74) at T2, with a t-value of 10.213 and a highly significant *p*-value (*p* = 0.000 **). Additionally, the time up and go test mean score decreased from 23.42 s (±3.05) at T1 to 21.50 s (±2.68) at T2, showing a t-value of 5.434 and a highly significant *p*-value (*p* = 0.000 **). Moreover, the Berg balance scale mean score increased significantly from 28.00 (±2.57) at T1 to 33.64 (±3.34) at T2, with a t-value of −14.593 and a highly significant *p*-value (*p* = 0.000 **). Similarly, the falls efficacy scale mean score increased from 19.28 (±3.12) at T1 to 22.64 (±2.70) at T2, with a t-value of −6.209 and a highly significant *p*-value (*p* = 0.000 **). Lastly, the modified Barthel index mean score increased from 35.64 (±5.03) at T1 to 39.50 (±2.92) at T2, with a t-value of −5.756 and a highly significant *p*-value (*p* = 0.000 **).

The control group’s functional outcomes at baseline and after 6 weeks were also analyzed in this study. The control group showed a significant decrease in the mean score for the five times sit-to-stand test from 16.57 s (±2.56) at T1 to 14.42 s (±2.13) at T2, with a t-value of 5.491 and a highly significant *p*-value (*p* = 0.000 **). Additionally, the time up and go test mean score decreased from 21.78 s (±2.15) at T1 to 20.35 s (±3.43) at T2, exhibiting a t-value of 3.333 and a significant *p*-value (*p* = 0.005 *). Moreover, the Berg balance scale mean score increased significantly from 29.21 (±5.17) at T1 to 32.50 (±3.81) at T2, with a t-value of −6.216 and a highly significant *p*-value (*p* = 0.000 **). The falls efficacy scale mean score also increased from 19.85 (±3.99) at T1 to 21.42 (±2.97) at T2, with a t-value of −2.705 and a significant *p*-value (*p* = 0.018 ). Lastly, the modified Barthel index mean score increased from 33.78 (±6.25) at T1 to 36.85 (±4.05) at T2, with a t-value of −3.466 and a significant *p*-value (*p* = 0.004 *).

### 3.3. Comparative Analysis between Groups Using the Amount of Change between Evaluations

Table 3 presents a comparative analysis between the experimental and control groups on the mean difference between baseline and 6-week assessments. The amounts of change in functional outcomes, along with the corresponding independent *t*-test results, are depicted. The experimental group exhibited a significantly greater improvement in the five times sit-to-stand test mean difference (3.92 ± 1.43 s) compared to the control group (2.14 ± 1.46 s), with a t-value of 3.259 and a highly significant *p*-value (*p* = 0.003 *). However, there was no significant difference between the groups for the time up and go test mean difference, with a t-value of 0.899 and a *p*-value of 0.377. Furthermore, the experimental group showed a significantly greater improvement in the Berg balance scale mean difference (−5.64 ± 1.44) compared to the control group (−3.28 ± 1.97), with a t-value of −3.599 and a highly significant *p*-value (*p* = 0.001 *). Similarly, the falls efficacy scale mean difference was significantly greater for the experimental group (−3.35 ± 2.02) compared to the control group (−1.57 ± 2.17), with a t-value of −2.250 and a significant *p*-value (*p* = 0.033). However, no significant difference was observed between the groups in the modified Barthel index mean difference, with a t-value of −0.707 and a *p*-value of 0.486.

In summary, the experimental group demonstrated significantly greater improvements in FTSST, BBS, and FES mean differences compared to the control group. No significant differences were found between the groups in TUG and MBI mean differences. These findings suggest that the intervention applied to the experimental group was more effective in enhancing balance maintenance and reducing fall risk compared to the control group.

## 4. Discussion

In this study, in order to investigate the effect of diagonal exercise using PNF on the functional activities of stroke patients, the study was conducted by dividing the experimental group receiving diagonal training and the control group receiving sagittal exercise. In the results of this study, statistically significant differences were found in all evaluation items representing functional activity in the post-evaluation compared to the pre-evaluation in all groups. In addition, the experimental group using PNF showed statistically significant differences in FTSST, BBS, and FES evaluation compared to the control group.

Humans have a complex anatomy that allows them to move in many directions. The most important type of movement is the diagonal movement, which is an important factor in maintaining correct posture, balance, and coordination. Diagonal movement occurs along the diagonal plane, which is a combination of the sagittal and frontal planes. These movements are essential for the functional use of the upper and lower extremities, including joint movements (extension, lateral flexion, and rotation), walking, activities of daily living, and sports [30].

Reduced diagonal movement is a common consequence of stroke and can significantly affect a patient’s functional ability. Reduced diagonal movement in stroke patients can lead to reduced voluntary movement, balance disorders, and gait disorders, which can affect the overall quality of life over time [31]. Reduced diagonal movement in stroke patients has a significant impact on upper extremity function, which limits their ability to perform basic self-care tasks such as dressing and grooming and may affect their ability to feed themselves [32]. The reduced lower extremity diagonal movement also affects the patient’s mobility. Decreased walking ability, which requires a complex interaction between sagittal and frontal movements, is a typical post-stroke symptom that increases the risk of falls and limits the ability and opportunities to engage in daily activities outside the home [33]. Fukata et al. (2021) investigated the effect of training to improve the sense of balance by sitting diagonally on an inclined surface in patients with low sedentary ability in the initial state of stroke patients [34]. In the results of the study, the experimental group, which trained by sitting on a surface inclined at 10 degrees, showed higher effects in FIST, SPV, and TIS compared to the control group. In conclusion, it was demonstrated that balance training applied on an inclined surface is effective in improving sitting ability and postural verticality in the diagonal plane.

Maintaining balance is an essential aspect of daily living that allows individuals to carry out various activities, such as walking, standing, and moving around, without falling or losing stability. However, stroke patients often experience impaired balance, which is a significant concern for their safety, mobility, and quality of life [4,18]. Balance is a complex process that involves the interaction of various systems in the body, including the sensory, perceptual, cognitive, and motor systems as well as the musculoskeletal system. To maintain balance, individuals must adapt to external stimuli by adjusting their posture and movements [35]. There are two main types of balance, static and dynamic. Static balance refers to the ability to stand still on a fixed surface without wobble or instability. On the other hand, dynamic balance is maintaining balance when the supporting surface moves or an external stimulus is present [36]. A stroke can cause neurological symptoms related to motor function, including muscle weakness and loss of sensation. As a result, it is not only difficult to maintain proper muscle tone and posture, but also the ability to maintain balance is reduced because abnormalities in movement control occur [4,18].

There are several factors that can weaken balance in individuals with hemiplegia due to stroke, including motor, visual, and sensory functions, cerebellar lesions, and vestibular dysfunction. As a result, stroke patients may experience decreased postural stability, asymmetry in weight distribution when standing, and reduced balance during dynamic standing [37]. Therefore, it is essential to include effective therapeutic interventions aimed at improving functional balance as part of a comprehensive rehabilitation plan for each stroke patient. This can help to address the specific balance deficits of each individual and promote a complete recovery. Strokes can damage sensory pathways in the brain, impairing proprioception. This can lead to poor balance control, reduced postural stability, and increased risk of falls, and impaired proprioception can also affect motor planning and execution, resulting in abnormal movement patterns and poor coordination [38]. Improving proprioception can improve balance control and mobility in stroke patients, and such training should include exercises that focus on improving sensory processing, body awareness, and motor control [39]. In addition, proprioception training can improve balance ability and reduce the risk of falls in stroke patients [40].

In this study investigating the effectiveness of diagonal exercise on balance ability in stroke patients, it was found that the experimental group who performed the exercise had a positive effect on the improvement of balance ability. The results of the study showed that the experimental group had a statistically significant increase in balance ability compared to the control group. The diagonal motion exercise was found to be more effective than the sagittal motion exercise in improving balance ability, as it involved movements related to functional activities that required the activation of multiple muscles involved in moving the joints of the upper and lower limbs. Furthermore, the diagonal motion exercise led to increased activity of the muscles related to the stability of the trunk, which is important for maintaining balance during functional activities. The exercise also improved the coordinated movement ability of the muscles around the joint, leading to an improvement in balance ability. In other words, diagonal training using PNF has demonstrated significant balance improvement on the FTSST and BBS compared to sagittal plane training for several reasons. Firstly, PNF training focuses on the integration of functional movements that mimic real-life activities. This approach targets multiple muscle groups and joints through diagonal patterns, thereby engaging the neuromuscular system more effectively than sagittal plane training, which focuses primarily on movements in a single plane. Secondly, the principles of PNF emphasize the importance of motor learning and coordination. By using techniques such as resistance, proprioceptive feedback, and alternating contractions of agonist and antagonist muscles, PNF training promotes the development of efficient and coordinated movement patterns. This leads to improved balance and functional performance on tests such as the FTSST and BBS. Thirdly, PNF training enhances proprioceptive feedback by stimulating muscle spindles and Golgi tendon organs. This increased sensory awareness allows for a better perception of joint position and muscle tension, leading to improved balance and stability during functional tasks. Another advantage of PNF training is that it targets muscles that are often underused or weak, such as the hip abductors and external rotators. Strengthening these muscles contributes to better balance and functional performance, as demonstrated by improvements in the FTSST and BBS.

However, in this study, no significant difference was found between the experimental group and the control group in the TUG test. The lack of significant difference between diagonal training using PNF and sagittal training on the TUG could be attributed to the characteristics of both diagonal and sagittal motions and the specific demands of the TUG test. Firstly, it is important to consider the movement planes involved in both types of motion. Diagonal motion using PNF focuses on diagonal and spiral movement patterns that integrate functional movements and target multiple muscle groups and joints. In contrast, sagittal motion primarily involves movements in a single plane, such as flexion and extension. The TUG test mainly assesses the ability to rise from a chair, walk a short distance, turn, and return to the chair, which predominantly involves sagittal plane movements. As a result, the diagonal motion using PNF may not have a direct impact on the specific components of the TUG test. Another factor to consider is the gravitational influence on the TUG test. The test requires the ability to maintain balance and control while standing and walking, which are significantly influenced by gravity. The PNF exercises were performed in a position that does not involve standing, such as a supine position; therefore, the training might not have had a direct impact on the participants’ ability to perform the TUG test effectively.

Falls are a common and serious problem for stroke patients, and fall prevention requires a multidisciplinary approach that includes patient risk assessment, environmental modifications, use of assistive devices, application of appropriate therapeutic interventions, and patient and caregiver education [41]. Stroke patients are particularly vulnerable to falls due to physical impairments such as muscle weakness, balance problems, and coordination difficulties. Since these falls can result in serious injuries such as fractures and head trauma, it is important to implement effective fall prevention methods [42].

Risk factors that cause falls are classified into intrinsic and extrinsic risk factors. Intrinsic risk factors are related to the decrease in physical function due to aging, and include age, lower extremity muscle strength, balance and walking ability, cognitive impairment, and chronic diseases. External risk factors are related to the environment and include seasons, places, times, and activities during falls [43,44]. Among internal and external factors, lower extremity muscle strength and balance are most closely related to falls. However, since falls are not caused by one or two factors but by a complex result of various factors, the more factors related to falls, the higher the risk of falls [35]. As a result of these falls, functional activity is reduced due to ischial bone fracture, soft tissue injury, and prolonged bed life, which consequently deteriorates physical, mental, and social well-being [45].

Many individuals who have experienced a fall develop a fear of falling again, which can lead to activity restriction. Research shows that over 80% of respondents who reported a fear of falling indicated that they became more cautious and less active after experiencing a fall [46]. A previous study investigated the fear of falling in the elderly and found that the older the elderly, the higher the fear of falling, and the fear of falling was higher in women than in men [47,48]. The fear of falling can lead to a reduction in physical activity and functional ability, which can decrease an individual’s independence and lead to a cycle of decreased physical ability that further increases the risk of falls. Additionally, fear of falling is closely related to actual falls, and the physical injuries and emotional distress caused by falls can significantly impact the daily lives of stroke patients [49]. Therefore, rehabilitation programs should include therapeutic interventions to address the fear of falling and reduce the risk of falls. By doing so, stroke patients can regain confidence in their ability to perform activities and improve their overall quality of life.

To prevent falls in stroke patients, a comprehensive treatment approach should be used. Stroke patients often experience reduced muscle flexibility and strength due to muscle hypertonia, spasm, changes in muscle connective tissue, and contractures, which can limit active movement and decrease overall balance control, increasing the risk of falls [50]. Therefore, appropriate intervention methods that can increase muscle strength and balance ability while also promoting controlled muscle movements are necessary to prevent falls in stroke patients. Currently, the clinical practice includes various intervention methods to prevent falls in stroke patients, including exercise therapy [41,45], robot therapy [15], electrical therapy [51], and drug therapy [52]. 

Among these methods, exercise therapy using PNF is a representative treatment method for fall prevention [17]. PNF therapy is a treatment method that uses sensory input to re-educate muscles. This therapy involves various techniques that activate muscle contraction, which can improve muscle strength and coordination [53,54]. Additionally, PNF therapy can improve balance control by increasing the activation of proprioceptive senses, and these senses provide the body with important information about joint position and movement, which can be used to improve overall balance and reduce the risk of falls [18].

In this study investigating the effects of diagonal motion exercise using the PNF technique on fear of falling in stroke patients, it was found that the experimental group who performed the diagonal exercise had a statistically significant decrease in fear of falling compared to the control group who performed the sagittal plane exercise. It is thought that the decrease in fear of falling is due to the increase in proprioception, muscle strength, and coordination of muscles around the joints that occurred through the oblique movement exercise using the PNF technique. These results suggest that diagonal exercise using the PNF technique can be an effective intervention to reduce the fear of falling in stroke patients by making them more confident in their ability to perform functional activities and maintain independence.

However, there are several limitations to this study. As a pilot study, the number of participants was small, and the evaluations were conducted only twice, as pre- and post-assessments. Most evaluations were not quantitative, and the intervention period was short, at only 6 weeks. Based on this study, it is hoped that future research will investigate the effects of diagonal motion exercise on a larger number of participants over an extended period to validate its effectiveness. Additionally, it is desired that future studies utilize quantitative methods for evaluating gait and balance abilities in stroke patients and conduct repeated assessments to observe changes in treatment outcomes.

## 5. Conclusions

Stroke is a serious condition that can have a significant impact on a patient’s quality of life and independence. Activities of daily living are often affected, leading to decreased self-esteem, increased depression, and reduced overall well-being. In order to improve the functional ability and quality of life of stroke patients, effective rehabilitation programs that focus on promoting motor function and self-reliance are essential. Diagonal motion exercise involves movements that are related to functional activities, which can improve strength, coordination, and overall motor control. The PNF technique used in diagonal exercise also improves proprioception and coordination of the muscles around the joints, which can increase confidence in performing functional activities and maintaining independence. This dissertation investigated the effectiveness of diagonal exercise using the PNF technique on improving balance ability and fear of falling in stroke patients. The findings suggest that diagonal motion exercise can be a highly effective intervention for improving dynamic balance ability and overcoming the fear of falling in stroke patients.

## Figures and Tables

**Table 1:** Homogeneity of general characteristics of subjects and pre-test.

	EP (n=14)	CO (n = 14)	*p*-Value
Age (years)	68.21 ± 3.01	67.07 ± 2.58	0.292
Weight (kg)	59.21 ± 5.47	61.28 ± 5.31	0.319
Height (cm)	156.92 ± 2.22	159.00 ± 4.20	0.155
FTSST (second)	17.78 ± 2.54	16.57 ± 2.56	0.220
TUG (second)	23.42 ± 3.05	21.78 ± 2.15	0.112
BBS (score)	28.00 ± 2.57	29.21 ± 5.17	0.439
FES (score)	19.28 ± 3.12	19.85 ± 3.99	0.677
MBI (score)	35.64 ± 5.03	33.78 ± 6.25	0.395

Abbreviations: Number (n); experimental group (EP); control group (CO); five times sit-to-stand test (FTSST); time up and go test (TUG); Berg balance scale (BBS); falls efficacy scale (FES); modified Barthel index (MBI). Notes: Data are reported as mean ± SD.

**Table 2:** Clinical outcomes of the experimental group (EP) and control group (CO) at baseline (T1) and 6 weeks (T2) assessments. Changes in functional outcomes and the corresponding paired *t*-test are depicted.

Variable	Group	T1	T2	t	*p*
FTSST (Second)	EP (n = 14)	17.78 ± 2.54	13.85 ± 2.74	10.213	0.000 **
	CO (n = 14)	16.57 ± 2.56	14.42 ± 2.13	5.491	0.000 **
TUG (Second)	EP (n = 14)	23.42 ± 3.05	21.50 ± 2.68	5.434	0.000 **
	CO (n = 14)	21.78 ± 2.15	20.35 ± 3.43	3.333	0.005 *
BBS (Score)	EP (n = 14)	28.00 ± 2.57	33.64 ± 3.34	−14.593	0.000 **
	CO (n = 14)	29.21 ± 5.17	32.50 ± 3.81	−6.216	0.000 **
FES (Score)	EP (n = 14)	19.28 ± 3.12	22.64 ± 2.70	−6.209	0.000 **
	CO (n = 14)	19.85 ± 3.99	21.42 ± 2.97	−2.705	0.018
MBI (Score)	EP (n = 14)	35.64 ± 5.03	39.50 ± 2.92	−5.756	0.000 **
	CO (n = 14)	33.78 ± 6.25	36.85 ± 4.05	−3.466	0.004 *

Abbreviations: Number (n); experimental group (EP); control group (CO); five times sit-to-stand test (FTSST); time up and go test (TUG); Berg balance scale (BBS); falls efficacy scale (FES); modified Barthel index (MBI). T1: baseline assessment; T2: 6 weeks assessment. Notes: Data are reported as mean ± SD, * *p* < 0.01; ** *p =* 0.000.

**Table 3:** Comparative analysis between groups on the mean of the difference between the baseline (T1) and 6 weeks (T2) assessments. Amounts of change in functional outcomes and the corresponding independent *t*-test are depicted.

Variable	EP (n = 14)	CO (n = 14)	t	*p*	Effect Size
FTSST (Second)	3.92 ± 1.43	2.14 ± 1.46	3.259	0.003 *	1.229
TUG (Second)	1.92 ± 1.32	1.42 ± 1.60	0.899	0.377	0.341
BBS (Score)	−5.64 ± 1.44	−3.28 ± 1.97	−3.599	0.001 *	1.364
FES (Score)	−3.35 ± 2.02	−1.57 ± 2.17	−2.250	0.033	0.850
MBI (Score)	−3.85 ± 2.50	−3.07 ± 3.31	−0.707	0.486	0.266

Abbreviations: Number (n); experimental group (EP); control group (CO); five times sit-to-stand test (FTSST); time up and go test (TUG); Berg balance scale (BBS); falls efficacy scale (FES); modified Barthel index (MBI). Notes: Data are reported as mean ± SD, * *p* < 0.01.

## Data Availability

The datasets generated during the current study are available from the corresponding author upon reasonable request.

## References

[1] Battaglini D., Robba C., Lopes da Silva A., Dos Santos Samary C., Leme Silva P., Dal Pizzol F., Pelosi P., Rocco P.R.M. (2020). Brain-Heart Interaction after Acute Ischemic Stroke. Crit. Care Lond. Engl..

[2] Campbell B.C.V., De Silva D.A., Macleod M.R., Coutts S.B., Schwamm L.H., Davis S.M., Donnan G.A. (2019). Ischaemic Stroke. Nat. Rev. Dis. Primer.

[3] Sivolap Y.P., Damulin I.V. (2019). [Stroke and depression]. Zh. Nevrol. Psikhiatr. Im. S. S. Korsakova.

[4] Li J., Zhong D., Ye J., He M., Liu X., Zheng H., Jin R., Zhang S.-L. (2019). Rehabilitation for Balance Impairment in Patients after Stroke: A Protocol of a Systematic Review and Network Meta-Analysis. BMJ Open.

[5] Stinear C.M. (2017). Prediction of Motor Recovery after Stroke: Advances in Biomarkers. Lancet Neurol..

[6] Lo Coco D., Lopez G., Corrao S. (2016). Cognitive Impairment and Stroke in Elderly Patients. Vasc. Health Risk Manag..

[7] Longley V., Hazelton C., Heal C., Pollock A., Woodward-Nutt K., Mitchell C., Pobric G., Vail A., Bowen A. (2021). Non-Pharmacological Interventions for Spatial Neglect or Inattention Following Stroke and Other Non-Progressive Brain Injury. Cochrane Database Syst. Rev..

[8] Denissen S., Staring W., Kunkel D., Pickering R.M., Lennon S., Geurts A.C., Weerdesteyn V., Verheyden G.S. (2019). Interventions for Preventing Falls in People after Stroke. Cochrane Database Syst. Rev..

[9] Mansfield A., Inness E.L., Mcilroy W.E. (2018). Stroke. Handb. Clin. Neurol..

[10] Tasseel-Ponche S., Delafontaine A., Godefroy O., Yelnik A.P., Doutrellot P.-L., Duchossoy C., Hyra M., Sader T., Diouf M. (2022). Walking Speed at the Acute and Subacute Stroke Stage: A Descriptive Meta-Analysis. Front. Neurol..

[11] Eraifej J., Clark W., France B., Desando S., Moore D. (2017). Effectiveness of Upper Limb Functional Electrical Stimulation after Stroke for the Improvement of Activities of Daily Living and Motor Function: A Systematic Review and Meta-Analysis. Syst. Rev..

[12] García-Rudolph A., Sánchez-Pinsach D., Salleras E.O., Tormos J.M. (2019). Subacute Stroke Physical Rehabilitation Evidence in Activities of Daily Living Outcomes: A Systematic Review of Meta-Analyses of Randomized Controlled Trials. Medicine.

[13] Rabinstein A.A. (2017). Treatment of Acute Ischemic Stroke. Contin. Minneap. Minn.

[14] Srithumsuk W., Chaleoykitti S., Jaipong S., Pattayakorn P., Podimuang K. (2021). Association between Depression and Medication Adherence in Stroke Survivor Older Adults. Jpn. J. Nurs. Sci. JJNS.

[15] Klamroth-Marganska V. (2018). Stroke Rehabilitation: Therapy Robots and Assistive Devices. Adv. Exp. Med. Biol..

[16] Le Danseur M. (2020). Stroke Rehabilitation. Crit. Care Nurs. Clin. N. Am..

[17] Guiu-Tula F.X., Cabanas-Valdés R., Sitjà-Rabert M., Urrútia G., Gómara-Toldrà N. (2017). The Efficacy of the Proprioceptive Neuromuscular Facilitation (PNF) Approach in Stroke Rehabilitation to Improve Basic Activities of Daily Living and Quality of Life: A Systematic Review and Meta-Analysis Protocol. BMJ Open.

[18] Nguyen P.T., Chou L.-W., Hsieh Y.-L. (2022). Proprioceptive Neuromuscular Facilitation-Based Physical Therapy on the Improvement of Balance and Gait in Patients with Chronic Stroke: A Systematic Review and Meta-Analysis. Life Basel Switz..

[19] Alahmari K.A., Silvian P., Ahmad I., Reddy R.S., Tedla J.S., Kakaraparthi V.N., Rengaramanujam K. (2020). Effectiveness of Low-Frequency Stimulation in Proprioceptive Neuromuscular Facilitation Techniques for Post Ankle Sprain Balance and Proprioception in Adults: A Randomized Controlled Trial. BioMed. Res. Int..

[20] Hindle K.B., Whitcomb T.J., Briggs W.O., Hong J. (2012). Proprioceptive Neuromuscular Facilitation (PNF): Its Mechanisms and Effects on Range of Motion and Muscular Function. J. Hum. Kinet..

[21] Davis D.S., Ashby P.E., McCale K.L., McQuain J.A., Wine J.M. (2005). The Effectiveness of 3 Stretching Techniques on Hamstring Flexibility Using Consistent Stretching Parameters. J. Strength Cond. Res..

[22] Moreira R., Lial L., Teles Monteiro M.G., Aragão A., Santos David L., Coertjens M., Silva-Júnior F.L., Dias G., Velasques B., Ribeiro P. (2017). Diagonal Movement of the Upper Limb Produces Greater Adaptive Plasticity than Sagittal Plane Flexion in the Shoulder. Neurosci. Lett..

[23] Smedes F., Giacometti da Silva L. (2019). Motor Learning with the PNF-Concept, an Alternative to Constrained Induced Movement Therapy in a Patient after a Stroke; a Case Report. J. Bodyw. Mov. Ther..

[24] van Dijk M.M., Meyer S., Sandstad S., Wiskerke E., Thuwis R., Vandekerckhove C., Myny C., Ghosh N., Beyens H., Dejaeger E. (2017). A Cross-Sectional Study Comparing Lateral and Diagonal Maximum Weight Shift in People with Stroke and Healthy Controls and the Correlation with Balance, Gait and Fear of Falling. PLoS ONE.

[25] Muñoz-Bermejo L., Adsuar J.C., Mendoza-Muñoz M., Barrios-Fernández S., Garcia-Gordillo M.A., Pérez-Gómez J., Carlos-Vivas J. (2021). Test-Retest Reliability of Five Times Sit to Stand Test (FTSST) in Adults: A Systematic Review and Meta-Analysis. Biology.

[26] Chan P.P., Si Tou J.I., Tse M.M., Ng S.S. (2017). Reliability and Validity of the Timed Up and Go Test With a Motor Task in People With Chronic Stroke. Arch. Phys. Med. Rehabil..

[27] Park S.-H., Lee Y.-S. (2017). The Diagnostic Accuracy of the Berg Balance Scale in Predicting Falls. West. J. Nurs. Res..

[28] Yardley L., Beyer N., Hauer K., Kempen G., Piot-Ziegler C., Todd C. (2005). Development and Initial Validation of the Falls Efficacy Scale-International (FES-I). Age Ageing.

[29] Ohura T., Hase K., Nakajima Y., Nakayama T. (2017). Validity and Reliability of a Performance Evaluation Tool Based on the Modified Barthel Index for Stroke Patients. BMC Med. Res. Methodol..

[30] Lee Y.-J., Liang J.N., Chen B., Aruin A.S. (2019). Characteristics of Medial-Lateral Postural Control While Exposed to the External Perturbation in Step Initiation. Sci. Rep..

[31] Rodríguez-Fernández A., Lobo-Prat J., Font-Llagunes J.M. (2021). Systematic Review on Wearable Lower-Limb Exoskeletons for Gait Training in Neuromuscular Impairments. J. Neuroeng. Rehabil..

[32] Chen J., Or C.K., Chen T. (2022). Effectiveness of Using Virtual Reality-Supported Exercise Therapy for Upper Extremity Motor Rehabilitation in Patients with Stroke: Systematic Review and Meta-Analysis of Randomized Controlled Trials. J. Med. Internet Res..

[33] Darekar A., Lamontagne A., Fung J. (2017). Locomotor Circumvention Strategies Are Altered by Stroke: II. Postural Coordination. J. Neuroeng. Rehabil..

[34] Fukata K., Amimoto K., Inoue M., Sekine D., Inoue M., Fujino Y., Makita S., Takahashi H. (2021). Effects of Diagonally Aligned Sitting Training with a Tilted Surface on Sitting Balance for Low Sitting Performance in the Early Phase after Stroke: A Randomised Controlled Trial. Disabil. Rehabil..

[35] Cuevas-Trisan R. (2017). Balance Problems and Fall Risks in the Elderly. Phys. Med. Rehabil. Clin. N. Am..

[36] Rubega M., Formaggio E., Di Marco R., Bertuccelli M., Tortora S., Menegatti E., Cattelan M., Bonato P., Masiero S., Del Felice A. (2021). Cortical Correlates in Upright Dynamic and Static Balance in the Elderly. Sci. Rep..

[37] Han P., Zhang W., Kang L., Ma Y., Fu L., Jia L., Yu H., Chen X., Hou L., Wang L. (2017). Clinical Evidence of Exercise Benefits for Stroke. Adv. Exp. Med. Biol..

[38] Chiaramonte R., Bonfiglio M., Leonforte P., Coltraro G.L., Guerrera C.S., Vecchio M. (2022). Proprioceptive and Dual-Task Training: The Key of Stroke Rehabilitation, A Systematic Review. J. Funct. Morphol. Kinesiol..

[39] Van Criekinge T., Truijen S., Schröder J., Maebe Z., Blanckaert K., van der Waal C., Vink M., Saeys W. (2019). The Effectiveness of Trunk Training on Trunk Control, Sitting and Standing Balance and Mobility Post-Stroke: A Systematic Review and Meta-Analysis. Clin. Rehabil..

[40] Cadore E.L., Rodríguez-Mañas L., Sinclair A., Izquierdo M. (2013). Effects of Different Exercise Interventions on Risk of Falls, Gait Ability, and Balance in Physically Frail Older Adults: A Systematic Review. Rejuvenation Res..

[41] Yang F., Lees J., Simpkins C., Butler A. (2021). Interventions for Preventing Falls in People Post-Stroke: A Meta-Analysis of Randomized Controlled Trials. Gait Posture.

[42] Vahlberg B., Cederholm T., Lindmark B., Zetterberg L., Hellström K. (2017). Short-Term and Long-Term Effects of a Progressive Resistance and Balance Exercise Program in Individuals with Chronic Stroke: A Randomized Controlled Trial. Disabil. Rehabil..

[43] Ambrose A.F., Paul G., Hausdorff J.M. (2013). Risk Factors for Falls among Older Adults: A Review of the Literature. Maturitas.

[44] Gazibara T., Kurtagic I., Kisic-Tepavcevic D., Nurkovic S., Kovacevic N., Gazibara T., Pekmezovic T. (2017). Falls, Risk Factors and Fear of Falling among Persons Older than 65 Years of Age. Psychogeriatr. Off. J. Jpn. Psychogeriatr. Soc..

[45] Gschwind Y.J., Kressig R.W., Lacroix A., Muehlbauer T., Pfenninger B., Granacher U. (2013). A Best Practice Fall Prevention Exercise Program to Improve Balance, Strength/Power, and Psychosocial Health in Older Adults: Study Protocol for a Randomized Controlled Trial. BMC Geriatr..

[46] Parry S.W., Bamford C., Deary V., Finch T.L., Gray J., MacDonald C., McMeekin P., Sabin N.J., Steen I.N., Whitney S.L. (2016). Cognitive-Behavioural Therapy-Based Intervention to Reduce Fear of Falling in Older People: Therapy Development and Randomised Controlled Trial—The Strategies for Increasing Independence, Confidence and Energy (STRIDE) Study. Health Technol. Assess. Winch. Engl..

[47] Sertel M., Aydoğan Arslan S., Tütün Yümin E., Demirci C.S., Tarsuslu Şimşek T. (2021). Investigation of the Relationship between Physical Activity, Kinesiophobia and Fear of Falling in Older Adults with Chronic Pain. Somatosens. Mot. Res..

[48] Toyoda H., Hayashi C., Okano T. (2022). Associations between Physical Function, Falls, and the Fear of Falling among Older Adults Participating in a Community-Based Physical Exercise Program: A Longitudinal Multilevel Modeling Study. Arch. Gerontol. Geriatr..

[49] Lin S., Wang C., Wang Q., Xie S., Tu Q., Zhang H., Peng M., Zhou J., Redfern J. (2022). The Experience of Stroke Survivors and Caregivers during Hospital-to-Home Transitional Care: A Qualitative Longitudinal Study. Int. J. Nurs. Stud..

[50] Chow J.W., Stokic D.S. (2023). The Contribution of Walking Speed versus Recent Stroke to Temporospatial Gait Variability. Gait Posture.

[51] Nevisipour M., Honeycutt C.F. (2022). Investigating the Underlying Biomechanical Mechanisms Leading to Falls in Long-Term Ankle-Foot Orthosis and Functional Electrical Stimulator Users with Chronic Stroke. Gait Posture.

[52] Westerlind E.K., Lernfelt B., Hansson P.-O., Persson C.U. (2019). Drug Treatment, Postural Control, and Falls: An Observational Cohort Study of 504 Patients with Acute Stroke, the Fall Study of Gothenburg. Arch. Phys. Med. Rehabil..

[53] Alexandre de Assis I.S., Luvizutto G.J., Bruno A.C.M., Sande de Souza L.A.P. (2020). The Proprioceptive Neuromuscular Facilitation Concept in Parkinson Disease: A Systematic Review and Meta-Analysis. J. Chiropr. Med..

[54] Arcanjo F.L., Martins J.V.P., Moté P., Leporace G., de Oliveira D.A., de Sousa C.S., Saquetto M.B., Gomes-Neto M. (2022). Proprioceptive Neuromuscular Facilitation Training Reduces Pain and Disability in Individuals with Chronic Low Back Pain: A Systematic Review and Meta-Analysis. Complement. Ther. Clin. Pract..

